# Large plants enhance aboveground biomass in arid natural forest and plantation along differential abiotic and biotic conditions

**DOI:** 10.3389/fpls.2022.999793

**Published:** 2022-10-13

**Authors:** Bai-Yu Yang, Arshad Ali, Ming-Shan Xu, Min-Sha Guan, Yan Li, Xue-Ni Zhang, Xue-Min He, Xiao-Dong Yang

**Affiliations:** ^1^ Department of Geography & Spatial Information Technology, Ningbo University, Ningbo, China; ^2^ Institute of Resources and Environment Science, Xinjiang University, Urumqi, China; ^3^ Forest Ecology Research Group, College of Life Sciences, Hebei University, Baoding, China; ^4^ Institute of East China Sea, Ningbo University, Ningbo, China

**Keywords:** big-sized trees effect, climate water availability, niche complementarity effect, scaling theory, soil fertility

## Abstract

Big-sized trees, species diversity, and stand density affect aboveground biomass in natural tropical and temperate forests. However, these relationships are unclear in arid natural forests and plantations. Here, we hypothesized that large plants (a latent variable of tall-stature and big-crown, which indicated the effect of big-sized trees on ecosystem function and structure) enhance aboveground biomass in both arid natural forests and plantations along the gradients of climate water availability and soil fertility. To prove it, we used structural equation modeling (SEM) to test the influences of large plants located in 20% of the sequence formed by individual size (a synthetical value calculated from tree height and crown) on aboveground biomass in natural forests and plantations while considering the direct and indirect influences of species diversity as well as climatic and soil conditions, using data from 73 natural forest and 30 plantation plots in the northwest arid region of China. The results showed that large plants, species diversity, and stand density all increased aboveground biomass. Soil fertility declined aboveground biomass in natural forest, whereas it increased biomass in plantation. Although climatic water availability had no direct impact on aboveground biomass in both forests, it indirectly controlled the change of aboveground biomass *via* species diversity, stand density, and large plants. Stand density negatively affects large plants in both natural forests and plantations. Species diversity positively affects large plants on plantations but not in natural forests. Large plants increased slightly with increasing climatic water availability in the natural forest but decreased in plantation, whereas soil fertility inhibited large plants in plantation only. This study highlights the extended generality of the big-sized trees hypothesis, scaling theory, and the global importance of big-sized tree in arid natural forests and plantations.

## Introduction

**Figure 1:**
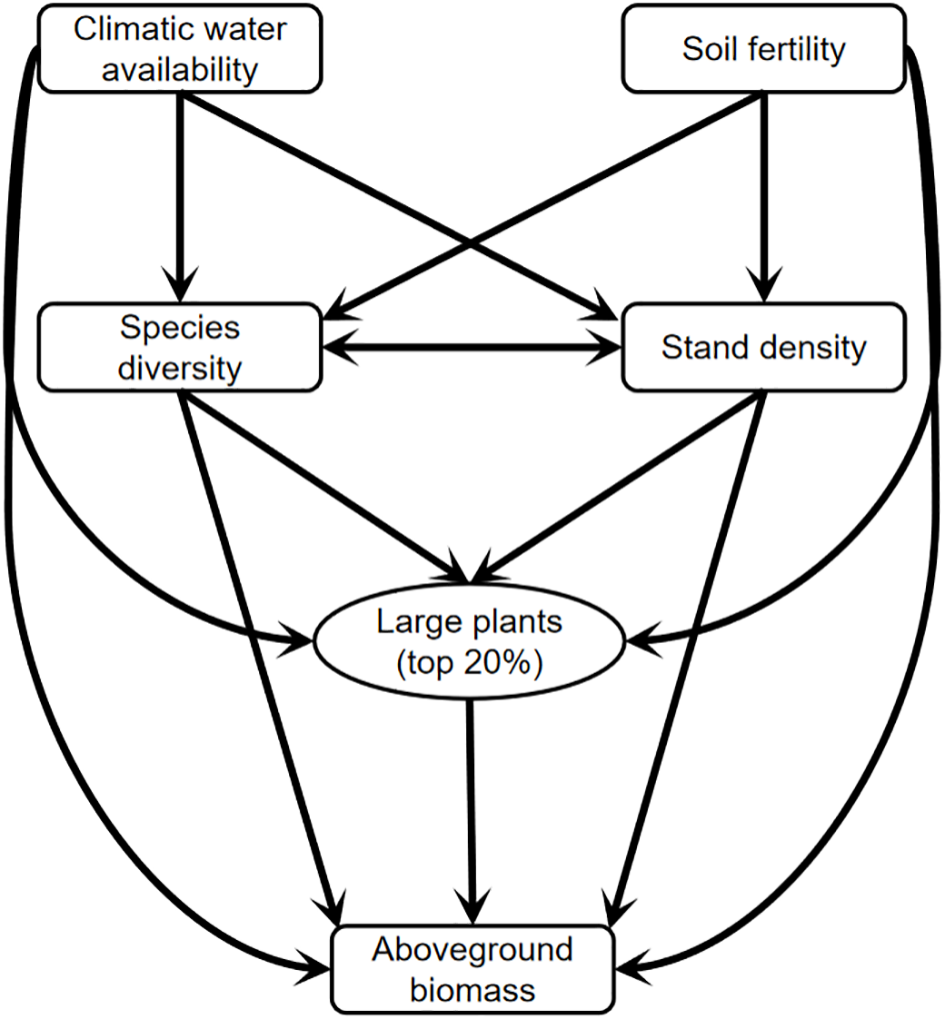
A conceptual model for testing the linkages among climatic water availability, soil fertility, large plants, species diversity, stand density, and aboveground biomass in natural forest and plantation of the arid temperate region in China.

How species diversity loss affects ecosystem functioning has sparked numerous concerns over the past three decades (Hector et al., 1999; Hooper et al., 2005; Huang et al., 2018). Evidence is mounting that a decline in species richness or diversity has a negative effect on plant productivity in grasslands (Tilman et al., 1996; Liang et al., 2016; van der Plas, 2019). Through this understanding, the niche complementarity hypothesis suggests that plant productivity or aboveground biomass increases with increasing plant species diversity because the coexistence of species can use the available resources more efficiently (Tilman et al., 1997; Loreau and Hector, 2001; Hooper et al., 2005). The selection effect hypothesis proposes that community productivity is often shifting with increasing species diversity due to the higher probability of productive species. In forest ecosystems, a considerable number of studies have demonstrated that large-diameter trees (big-sized trees) contribute disproportionally to aboveground biomass at either the individual or stand level (Clark and Clark, 1996; Lutz et al., 2012; Bastin et al., 2015). In addition to the influence of big-sized trees on carbon enhancement, species diversity and stand structure have been tested to promote forest productivity or aboveground biomass through the niche complementarity mechanism (Yachi and Loreau, 2007; Mensah et al., 2018). Although aboveground biomass shifting with big-sized trees and species diversity has been well documented in natural tropical and temperate forest ecosystems with high diversity and strong resistance (Ali et al., 2019b; Yuan et al., 2021), these responses and mechanisms in species-poor arid temperate forests (particularly in arid natural forest and plantation) remain largely understudied (**Figure 1**).

In global forests and woodlands, big-sized trees dominate above- and belowground carbon storage and reallocation processes, as well as provide abundant habitats for vertebrates, invertebrates, and microorganisms through their individual specific traits, such as large diameter, tall height, and big crown (Luyssaert et al., 2008; Lindenmayer et al., 2012; Lutz et al., 2012). More specifically, the scaling theory suggests that a few big-sized trees contribute a great quantity of the aboveground biomass (Clark and Clark, 1996; Slik et al., 2013). A study from moist tropical forests found that 1.5% large-diameter trees explain more than 50% of aboveground biomass variation at a regional scale (Bastin et al., 2015). Moreover, a global study of 48 primary and secondary forests indicated that the largest 1% of trees comprised 50% of aboveground live biomass, and the diameter threshold of big-sized trees has a positive effect on aboveground biomass (Lutz et al., 2018). However, what kind of role the big-sized trees play in species diversity-aboveground biomass is still unclear. A recent study of tropic forests revealed that big-sized trees have a strong positive effect on forest carbon storage in the natural forest but a negligible effect in plantations, which proved that the regulation of big-sized trees in species diversity-aboveground biomass might be dependent on vegetation types (Mensah et al., 2020). Compared to natural forests, plantations characterized by lower species diversity consisted of fast-growing woods (Erskine et al., 2006; Kelty, 2006; Subedi et al., 2012). There are unpredictable feedback differences in carbon sequestration to species diversity and large plants (a latent variable of tall-stature and big-crown of big-sized trees, which indicate the effect of big-sized trees on ecosystem function and structure) between natural forest and plantation. Nevertheless, we know little about the joint effects of species diversity and large plants on aboveground biomass in plantations.

Aboveground biomass responds differently to species diversity with various stand structures in natural forests, such as stand density (Forrester et al., 2013; Yoshida et al., 2017; Ouyang et al., 2019). Most studies assume that stand density promotes carbon sequestration *via* the complementarity of crown light resources (Vitamin, 2015; Forrester et al., 2018). Nevertheless, there is a great difference between the stand density of plantations and natural forests. Previous studies demonstrated that higher stand density has a negligible effect on aboveground biomass in plantations due to asymmetric resource competition (Poorter et al., 2012; Condés et al., 2013; Ma et al., 2021). Furthermore, competition has been confirmed to cause a negative density dependence, which would promote species diversity (LaManna et al., 2017), and thus, a shifting species diversity-aboveground biomass relationship would occur in plantations among various stand densities. Some studies have shown that stand structure and large plants regulate aboveground biomass jointly in natural forests (Ali et al., 2019a; Ouyang et al., 2019). Yet, we do not know whether the joint effects of stand density, species diversity, and large plants on aboveground biomass exist in plantations.

In natural forests and plantations, the effects of large plants and species diversity on aboveground biomass could be mediated by the variability of climate and soil conditions (Zhang and Chen, 2015; Ali et al., 2019b; Li et al., 2020). Studies have often shown that climatic water availability rather than soil fertility plays a major role in determining aboveground biomass directly and indirectly *via* large plants and species diversity in natural forests (Ali et al., 2019b), but these mechanisms are yet to be understood in plantations. Furthermore, a study focusing on natural shrub forests in arid regions demonstrated that increasing climatic water availability weakens the response of biomass to species diversity and density because of strong interspecific competition between drought-sensitive species (Guo et al., 2019). However, several studies in tropical and subtropical natural forests found that soil fertility increased aboveground biomass *via* large plants (Ali et al., 2019a; Ouyang et al., 2019). These studies suggest that the responses of aboveground biomass to biotic factors are shifting with climate and soil conditions among different ecosystems. Therefore, to better understand and predict carbon sequestration, we need to consider species diversity, large plants, and stand density under various climate and soil conditions in natural forest and plantation ecosystems.

Arid ecosystems cover more than 41% of the global land surface and are one of the most frangible biosystems to climate change and human activities due to lower biodiversity (Reynolds et al., 2007). A double warming tendency over arid regions than humid areas has been confirmed because of the reduced carbon sequestration (Huang et al., 2016). Numerous public efforts have been made to slow down the expansion rates of environmental degradation (i.e., desertification and desert expansion), such as afforesting plantations (Raich and Tufekcioglu, 2000; Paul et al., 2002; Piao et al., 2009). However, as mentioned above, there are many unknowns about the response of carbon storage to species diversity, large plants, and stand density in arid regions. Consequently, we aim to evaluate the relative effects of large plants, species diversity, and stand density on aboveground biomass in arid natural forests and plantations under climate and soil conditions in this study (**Figure 1**). We expect that (1) large plants play a central role in driving aboveground biomass in natural forest and plantation, whereas species diversity and stand density are of additional importance; (2) large plants and species diversity do not maintain each other in both natural forest and plantation, and hence, both of them play an independent role in driving aboveground biomass; (3) climatic water availability and soil fertility regulate species diversity, large plants, stand density, and aboveground biomass in natural forest and plantation through several underlying ecological mechanisms.

## Materials and methods

### Study area and forest inventory

The study area is located in the northwest arid region of China (31° 42′–53° 23′ N, 73° 40′–126° 04′ E), and it includes five provinces (Xinjiang, Gansu, Inner Mongolia, Shaanxi, and Ningxia). The total area is about 3.55 million square kilometers, accounting for about 78% of the arid area of China. The study area belongs to a typical arid temperate continental climate, with an annual average rainfall ranging from 75 to 557 mm, an annual average evaporation ranges 700 to 2,300 mm, and an average temperature ranging from −1.5°C to 9.6°C (Chen et al., 2016; Chen et al., 2017).

**Figure 2:**
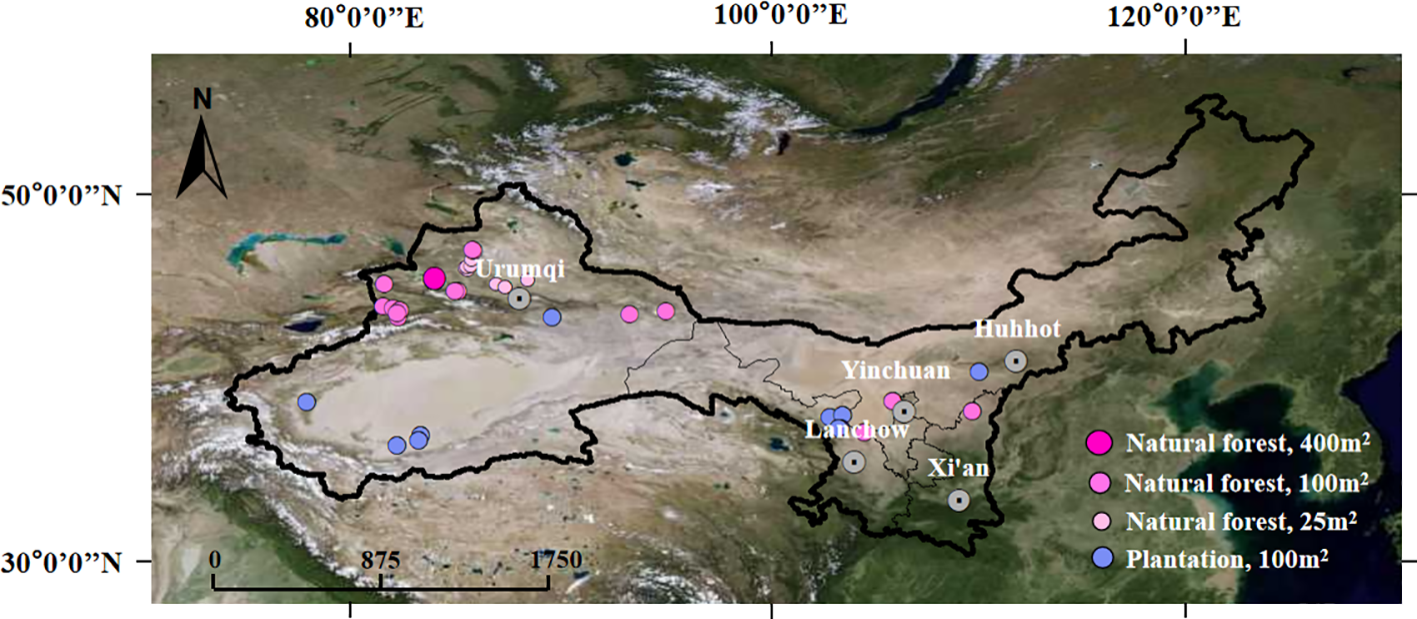
Sampling plot locations in the northwest arid region of China.

In this study, 103 plots (73 natural forest and 30 plantation plots, **Figure 2**) with sample areas equaling 25 and 400 m^2^ were investigated haphazardly in the northwest arid region of China from June to September 2019. According to previous studies, 25 and 100 m^2^ were the minimum sampling areas of shrub and forest communities, respectively, which covered the main changes in species and plant community composition in the arid ecosystem (Chen et al., 2017; Guo et al., 2019). The influence of the total study area on the sampling intensity was not considered in this study, because most arid areas in northwest China are deserts, while plant community types and species in other areas are few and have an uneven distribution (Guo et al., 2019). In the sampling process, we first considered plant communities and included all community types as much as possible. Meanwhile, we haphazardly set up sample points. However, the distance between sampling points was expanded largely to ensure that information contained by different points overlapped less.

In each plot, tree height, diameter at breast height (DBH, 1.3 m from the ground), basal diameter (0.2 m from the ground), and crown width of all woody plants were determined by using a Vertex meter (Vertex-IV, Haglöf Haglof, Dalarna, Sweden) and a tape, and a meter stick, respectively. Crown width was calculated as the average diameter of the east-west and north-south cross sections of the crown using a meter stick. Crown area was estimated using the equation (π × *D*_east-west_ × *D*_north–south_)/4. Chinese Flora Database (http://foceflora.cn/) was used to identify species at the taxonomic level.

Natural forest plots included 2,915 individuals belonging to 21 species, 20 genera, and 11 families. *Haloxylon ammodendron*, *Populus euphratica*, *Tamarix chinensisare*, and *Caragana Korshinskii* are the common species in arid natural forests. Plantation plots included 780 individuals belonging to 14 species, 12 genera, and nine families. The frequent species are *Haloxylon ammodendron*, *Ulmus glaucescens*, and *Elaeagnus angustifolia.*

### Aboveground biomass

We estimated the aboveground biomass of individual plants based on the species-specific allometric equations, which were based on the combinations of plant height, diameter, and crown dimensions (**Supplementary Table S1**). Total aboveground biomass is the sum of the aboveground biomass of all individual plants within each plot, which is then converted to a mega gram per hectare (Mg ha^−1^).

### Stand density, species diversity, and big-sized trees

Based on the forest inventory data, stand density was quantified as the total number of individuals per plot, which was then converted to stems per hectare. The Shannon–Wiener index was calculated to represent species diversity for each plot. In the forest community, a few big-sized trees could occupy the vast majority of the aboveground biomass (Slik et al., 2013; Bastin et al., 2015; Lutz et al., 2018). However, there is no universal definition for big-sized trees, but the general understanding is that big-sized trees vary among ecosystems (Lutz et al., 2018). For example, trees with a DBH of ≥20 cm in a cold coniferous forest, a DBH of ≥60 cm in temperate deciduous forests, and a DBH of ≥100 cm in tropical forests are generally defined as big-sized trees (Lutz et al., 2012). Still, different abiotic and biotic conditions may limit the threshold size for big-sized trees in different forest types and statuses. However, many woody plants have no DBH due to their short stature in arid regions (Guo et al., 2019). Precious studies have indicated that big-sized trees could be defined based on big-sized stature, for instance, plant height and crown (Lutz et al., 2018; Ali et al., 2019a). In order to characterize the big-sized trees, we used the entropy weight method (EWM) to calculate the weight of plant height and crown, then got the integrative values (58.62% stdheight + 41.38% stdcrown), which were then used to define the top 20% large trees within each plot. The proportion of the aboveground biomass of individuals in the top 1%, 5%, 10%, and 20% in the ranking position to the aboveground biomass was analyzed, respectively. In arid regions, especially in arid desert regions, severe environment makes the vast majority of individuals in sampling plots to be shrub species. Also, the effect of severe environment on plant growth results in small abundance in sampling plots. All big-sized trees might not be involved in the top 1%~10% of the ranking position. Therefore, the top 20% of individuals in the rank of DBH or base diameter were selected as the big-sized trees in this study. Our results indicated that there were 17 and 10 large plant species in natural forests and plantations, respectively (for more details, see **Supplementary Table S2**).

As suggested by Ali et al. (2019a), the influence of the top 20% of individuals in the rank of DBH or base diameter on forest structure and function was defined as the effect of the big-sized tree. It was a latent variable, represented by the “large plants,” and composed of tree height and crown area in this study. The internal reason for this design was that the tree height and crown area of big-sized trees have a greater impact on ecosystem processes, such as light acquisition, interspecific competition, and shading, in the forest compared with DBH and basal diameter. After that, the ratio of the sum of the top 20% of individual biomass to the total biomass was calculated to verify the existence of large plants in arid regions.

### Climatic water availability and soil fertility

To explore the influence of climatic water availability and soil fertility on the relationships among species diversity, stand density, large plants, and aboveground biomass, the annual climatic aridity index (CAI; mean annual precipitation/mean annual potential evapotranspiration) of each plot was downloaded from CGIAR-CSI (Trabucco and Zomer, 2009) to represent climatic water availability. Higher values of the aridity index represent the higher water content available for plant growth. The soil cation exchange capacity (CEC) of topsoil (0–30 cm) and subsoil (30–100 cm) were then obtained from Harmonized World Soil Database (FAO et al., 2012). Here, we used the mean soil CEC of topsoil and subsoil to define the soil fertility (Ali et al., 2019a).

### Statistical analyses

**Figure 3:**
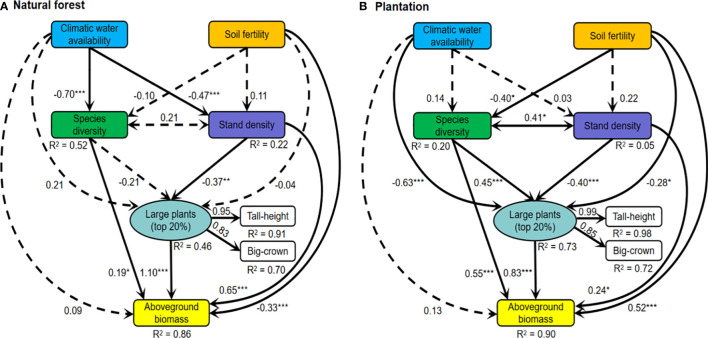
Structural equation models for linking climatic water availability, soil fertility, large plants (a latent variable of tall stature and big crown; indicated the influences of big-sized trees on ecosystem function and structure), species diversity, stand density, and aboveground biomass in **(A)** natural forest and **(B)** plantation of the arid temperate region in China. Solid lines represent significant paths (*p* < 0.05), while dashed lines indicate nonsignificant paths (*p* > 0.05). The confidence degree of the relationship between two paired variables at less than ^*^0.05, ^**^0.01, and ^***^0.001, respectively. For each path, the standardized regression coefficient is shown (**Supplementary Tables S3**, and **S4**). Model-fit statistics for **(A)**: comparative fit index (CFI) = 0.953, goodness-of-fit index (GFI) = 0.941, and the standardized root mean square residual (SRMR) = 0.052; **(B)**: CFI = 0.911, GFI = 0.941, SRMR = 0.055.

The structural equation model (SEM) was used to test the conceptual model for linking climatic water availability, soil fertility, species diversity, stand density, large plants (a latent variable of tall-height and big-crown, indicating the influences of big-sized trees on forest structure and function) and aboveground biomass in arid natural forest and plantation (**Figure 1**). The degree of model fit was evaluated by using the following three indicators: standardized root means square residual (SRMR <0.08), comparative fit index (CFI >0.90), and goodness-of-fit index (GFI >0.90). We also employed the Wald statistic test to assess the significance of each hypothesized pathway in SEM (*p* < 0.05). We used a maximum estimator with standard errors and scaled statistics to estimate standardized coefficients. Direct, indirect, and total effects were estimated by using standardized coefficients. After testing the SEM, we calculated the relative contribution of each predictor variable (i.e., climatic water availability, soil fertility, species diversity, stand density, large plants) to explain variance in aboveground biomass (Ali et al., 2019a). As suggested by Grace et al. (2016) and Yuan et al. (2021), the relative contribution of each predictor referred to its proportion in explaining the variation of AGB (total variance) among all sampling plots. To be specific, in the SEM models, the total AGB variance was evaluated from two stratifications: (1) the direct contribution of each predictor to the total AGB variance. According to the variance partition of the multiple regression, the total AGB variance was reduced to the part that could be explained by each predictor (regression sum of the square) and the other part that could not be explained by them (residual sum of variance). In the first part, the proportion of each predictor in explaining the total AGB variance was considered its contribution and (2) the indirect contribution of each predictor to the total AGB variance *via* mediator variables. For each pathway (e.g., **Figure 3**), the variance partition of the binary regression was used to calculate the contributions of the predictor to the total variance of the mediator variable, and the latter to the total AGB variance. The product of these two contributions was taken as the indirect contribution of the predictor to the total AGB variance. The sum of the direct and indirect contributions at two stratifications was the total contribution of each predictor to the change in AGB among all samples. The SEM was implemented using the *lavaan* package (Rosseel, 2012) in R 3.6.0 (R Core Team, 2020).

Before the analysis, we performed a natural logarithmic transformation of the data to meet the requirements of normality and linearity. In order to validate the results from SEM, we also performed simple linear regression for each pathway in SEM. A summary of the variables used in the analyses is presented in **Table 1**.

**Table 1:** Summary of the variables, from 73 natural forest plots and 30 plantation plots, was used in this study.

Forest type	Variable	Unit	Mean	SE	Minimum	Maximum
Natural forest	Top 20% tall height	m	5.58 a	1.01	0.73	41.00
	Top 20% big crown	m^2^	8.00 a	1.25	0.19	59.96
	Hs	#	0.84 a	0.06	0.00	1.66
	SD	N ha^−1^	3,859.93 a	324.15	800.00	18,400.00
	CAI	%	19.32 a	1.74	11.01	86.37
	Soil fertility	cmol kg^−1^	12.19 a	0.58	5.00	28.00
	AGB	Mg ha^−1^	27.02 a	7.00	0.03	353.28
	AGB proportion of 20% big-sized trees	Percentile	59.25 a	3.91	3.56	99.59
Plantation	Top 20% tall height	m	2.89 a	0.24	0.53	5.37
	Top 20% big crown	m^2^	4.34 a	0.48	0.61	12.17
	Hs	#	0.44 b	0.07	0.00	1.06
	SD	N ha^−1^	2,583.33 b	162.40	800.00	7,500.00
	CAI	%	11.64 b	1.94	1.23	33.43
	Soil fertility	cmol kg^−1^	12.00 a	0.70	6.00	26.00
	AGB	Mg ha^−1^	16.62 a	2.98	0.20	88.92
	AGB proportion of 20% big-sized trees	Percentile	39.66 b	4.10	7.82	97.53

Hs, species diversity; SD, stand density; CAI, climatic aridity index; AGB, aboveground biomass. All variables presented here are nontransformed "#" means that the index (Hs) has no unit (i.e., original data). Natural logarithm transformed values were used in the statistical analyses. Lowercase letters indicate significant differences between natural forest and plantation.

## Results

### Differences in the drivers of aboveground biomass between natural forest and plantation

Based on the statistical results of 73 natural forest and 30 plantation plots, our study found that climatic water availability, species diversity, stand density, and aboveground biomass of 20% of the big-sized trees in the natural forest were significantly higher than in plantations. There were no significant differences in soil fertility between natural forest and plantation. Specifically, the mean of aboveground biomass in natural forest (27.02 ± 7.00 Mg ha^−1^/mean ± SE) was higher than that of plantation (16.62 ± 2.98 Mg ha^−1^/mean ± SE). The top 20% of individuals, based on the rank of integrative values, hold 59.25% of total aboveground biomass in natural forest, whereas they account for 39.66% of total aboveground biomass in plantations (**Table 1**). The standard deviation in the proportion of the top 20% of individuals to total aboveground biomass in natural forest (0.33) was obviously higher than plantation (0.22) (**Table 1**).

### SEMs: What determines aboveground biomass directly and indirectly in natural forest and plantation

The SEMs for natural forest and plantation showed that large plants, species diversity, and stand density had significant positive direct effects on aboveground biomass (**Figure 3**). Stand density declined for large plants directly in both natural forest and plantation (*r*_NA_ = −0.37; *r*_PL_ = −0.40). Species diversity had a negligible direct effect on large plants in the natural forest but a significant positive direct effect on the plantation (*r*_NA_ = −0.21; *r*_PL_ = 0.45). Climatic water availability had a negligible direct effect on aboveground biomass in natural forest and plantation (*r*_NA_ = 0.09; *r*_PL_ = 0.13), while soil fertility directly decreased aboveground biomass in natural forest but increased in plantation (*r*_NA_ = −0.33; *r*_PL_ = 0.52). Climatic water availability had a negative effect on species diversity and stand density directly in natural forest (*r*_NA_ = −0.70; *r*_PL_ = −0.47) but negligible direct effects in plantation (*r*_NA_ = 0.14; *r*_PL_ = 0.03). Soil fertility exerted a negligible influence on species diversity in the natural forest but decreased species diversity directly in plantation (*r*_NA_ = −0.10; *r_PL_* = −0.40). Large plants increased slightly with an increase in climatic water availability in natural forest but decreased in plantation (*r*_NA_ = 0.21; *r*_PL_ = −0.63), whereas soil fertility inhibited large plants in plantation only (*r*_NA_ = −0.04; *r*_PL_ = 0.28; **Figure 3**).

**Figure 4:**
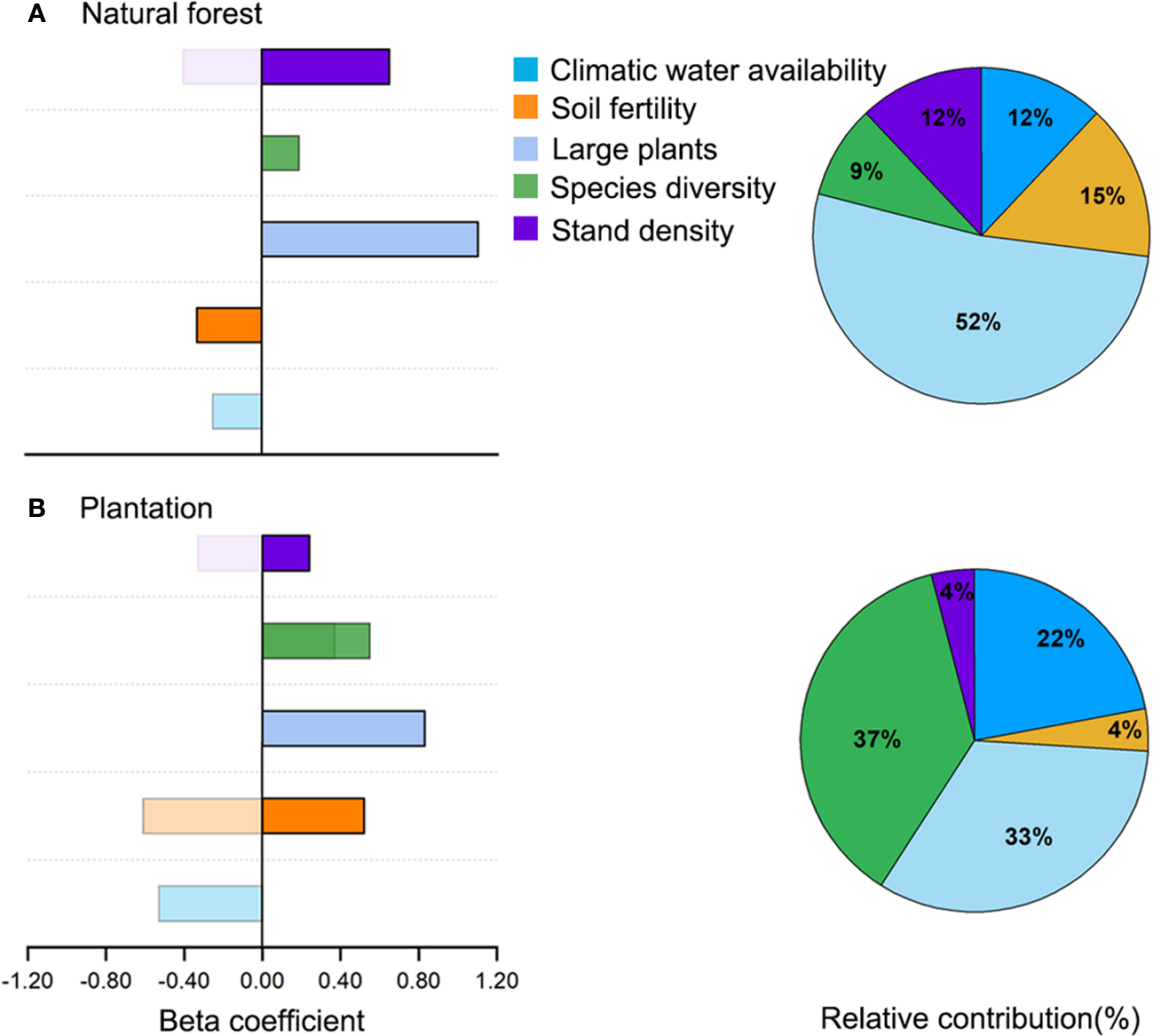
Total relative contribution (**A**; pie charts) of predictors to aboveground biomass and comparison of direct and indirect effects (**B**; bar charts) of predictors on aboveground biomass in natural forest and plantation. Solid-filled color represents direct effect, whereas pattern-filled color represents indirect effect.

The comparative analysis of direct and indirect effects showed that large plants possessed a strong positive direct effect on aboveground biomass than species diversity and stand density in natural forest, while species diversity possessed stronger positive direct and indirect effects on aboveground biomass than large plants and stand density in plantation (**Figures 3**, **4A**). Stand density possessed indirect negative effects on aboveground biomass *via* large plants in both natural forests and plantations. Species diversity possessed an indirect positive effect on aboveground biomass *via* large plants in the plantation but a negligible indirect effect in natural forests (**Figures 3**, **4A**). Soil fertility possessed a strong indirect negative effect on aboveground biomass *via* species diversity and large plants in the plantation but a negligible indirect effect in natural forests. Climatic water availability possessed a strong indirect effect on aboveground biomass *via* stand density, species diversity, and large plants in natural forests and to a little extent in plantations (**Figures 3**, **4A**).

The comparative analysis of the relative contribution (based on total effect or direct effect if there was no indirect effect) of drivers of aboveground biomass showed that, in natural forests, large plants explained higher variation in aboveground biomass followed by soil fertility, species diversity, climatic water availability, and stand density (**Figure 4B**). With respect to the plantation, species diversity explained higher variation in aboveground biomass, followed by large plants, climatic water availability, soil fertility, and stand density (**Figure 4B**). Bivariate relationships for each hypothesized path in natural forest and plantation are shown in **Supplementary Figures S3**, **S4**, respectively.

## Discussion

In this study, we explored the relative effects of large plants, species diversity, and stand density on aboveground biomass along climate and soil gradients in natural forests and plantations in an arid region. We found that large plants and species diversity increased aboveground biomass directly in natural forests and plantations, hence providing theoretical support to niche complementarity and big-sized trees effects (Morin et al., 2011; Ali et al., 2019a). However, these positive effects were different in plantations and natural forests, which might be due to the differential mechanisms and direct effects of climatic water availability and soil fertility on aboveground biomass, as well as indirect effects *via* large plants, species diversity, and stand density (Zhang and Chen, 2015; Ali et al., 2019b; Ouyang et al., 2019; Li et al., 2020). In sum, our results suggest that the small portion of biotic drivers (i.e., large plants, species diversity, and stand density) enhance aboveground biomass through similar mechanisms whereas the small portion of abiotic drivers regulates aboveground biomass through opposing mechanisms in natural forest versus plantation (van der Sande et al., 2017; Mensah et al., 2020; Wu et al., 2020).

Large plants increased aboveground biomass in both natural forests and plantations, providing the first evidence of the generality of the big-sized trees hypothesis as well as to scaling theory and the global importance of big-sized trees (Clark and Clark, 1996; Slik et al., 2013; Bastin et al., 2015; Lutz et al., 2018; Ali et al., 2019a). The strong positive effect of large plants on carbon sequestration in natural forests and plantations is because big-sized trees modulate the above- and belowground resource allocation *via* luxuriant branches and fine roots which has positive feedback on carbon storage (Luyssaert et al., 2008; Lutz et al., 2012). More specifically, we found that the taller and bigger crown plants dominate (i.e., top 20%) 59.25% and 39.66% of the biomass in a plot, respectively. The natural forests have a higher increase in aboveground biomass than plantation. These result is consistent with a recent local-scale study that showed that large plants have a stronger positive influence on aboveground biomass in natural forests than mixed plantations (Mensah et al., 2020). The change in the effect of large plants between natural forest and plantation largely results from the similar stand age in the plantation, which, against the generation of big-sized trees, further leads to a weaker big-sized tree effect (Ruiz-Jaen and Potvin, 2011; Subedi et al., 2012; Ouyang et al., 2019). Interestingly, we found no relationship between species diversity and large plants in natural forests but found a positive relationship in plantations. In natural forests, there is no relationship between species diversity and large plants, which might be further attributable to the environmental filtering effect (Morin et al., 2018). Drought-sensitive species and near-drought–resistant neighbors tend to aggregate in arid regions after environmental filtering (Guo et al., 2019; Wright et al., 2021), indicating that diverse community compositions may buffer multiple biotic interactions, such as the influence of species diversity on large plants. The positive relationship in the plantation is the result of niche complementarity, but we found a negative relationship between large plants and stand density in both natural forest and plantation. These results point to a competitive exclusion mechanism in the natural forest, whereas niche complementarity among species of dominant functional group in plantations (Yachi and Loreau, 2007; Subedi et al., 2012; Forrester et al., 2013; Liu et al., 2020), and thus lead to species competition due to big-sized trees in different ways, resulting in a reduction in stand density directly and aboveground biomass indirectly (van der Sande et al., 2017; Ouyang et al., 2019).

Aboveground biomass in the natural forest and plantation was mainly explained by large plants and species diversity, even though the relative contribution of the drivers varied due to the indirect effects in some cases. This pattern indicates species diversity and large plants may govern carbon sequestration in various ecosystems (Ruiz-Jaen and Potvin, 2011; Mensah et al., 2020). In plantations, species diversity promotes aboveground biomass through facilitating abiotic and biotic conditions simultaneously, for instance, light interception and seed dispersion (Binkley et al., 2010; Nagaike et al., 2012). In a natural forest, the positive relationship between species diversity and aboveground biomass was expected due to the complementarity effect, particularly in water availability complementarity (Guo et al., 2019; Wright et al., 2021; Yang et al., 2022). Previous studies showed that the positive facilitation between different plant species was common in arid regions, for instance, the hydraulic redistribution of soil water (Yu et al., 2013; Guo et al., 2019). Thus, deep-rooting species (such as *Populus euphratica* and *Tamarix chinensis*) would lift groundwater or deep soil water to the soil surface for the survival of other species (e.g., *Kali collinum* and *Alhagi sparsifolia*). However, the resource-use complementarity mechanism might have influenced aboveground biomass differentially in natural forests and plantations, probably due to the contrasting influences of resource supply patterns on species diversity.

Stand density has a strong positive effect on aboveground biomass in both natural forests and plantations. This similarity may be due to the resource complementarity effect, which means higher stand density intensifies the positive interspecies interactions that drive plants to make effective utilization of light, water, and soil nutrients (Morin et al., 2011; Forrester et al., 2018). However, stand density also had an indirect negative effect on the aboveground biomass of natural forest and plantation *via* large plants, indicating stand density may regulate the aboveground biomass in both natural forest and plantation by suppressing the dominant species, such as big-sized trees (Boyden et al., 2005; Subedi et al., 2012; Ouyang et al., 2019). Previous studies suggested that the direct and indirect effects of stand density on aboveground biomass are related to stand age, stand structure, functional diversity, and ecosystem types (Paquette and Messier, 2011; Guo and Ren, 2014; Yuan et al., 2018; Ouyang et al., 2019). For example, in subtropical forests, the positive regulation of stand density on aboveground biomass would be stronger with the increase of stand age. These results indicate the importance of stand density on aboveground biomass *via* different pathways in natural forests and plantations. However, our results differed from those of previous studies, which high densities result in lower biomass due to increased mortality from competition (Fernandez Tschieder et al., 2012; Sun et al., 2018). The potential reason for our results may be the complementarity effect of the resource. Two reasons may be explained for it: (1) the influences of stand closure or stand age (Guo and Ren, 2014; Ouyang et al., 2019): aboveground biomass decreased as stand density increased due to intensive sapling competition before stand closure. After stand closure, aboveground biomass shows an upward tendency with the increase of stand density because of resource complementarity; and (2) the influences of functional diversity (like RaoQ or CWM trait) (Yuan et al., 2018; Yuan et al., 2021): higher density may lead to lower productivity when the community consists of the same functional species, such as acquisitive species. On the contrary, it would increase productivity if the community had a higher functional diversity.

Climatic water availability had a negligible positive effect on aboveground biomass in both natural forest and plantation, whereas soil fertility directly increased aboveground biomass in plantation only, indicating that plant physiological and metabolic processes are directly influenced by the length of the growing season and nutrient availability, but the directions of these influences are context-dependent (Poorter et al., 2017; Ali et al., 2019a). However, it is also expected that species diversity, large plants, and stand density might be spatially structured and might be driven in part by abiotic factors differentially in natural forests and plantations (Van et al., 2017; van der Sande et al., 2017; Wang et al., 2017; Wu et al., 2020). As such, we found that climatic water availability and soil fertility influenced species diversity, large plants, and stand density through opposing mechanisms in natural forest and plantation, and hence differentially determined aboveground biomass indirectly *via* biotic factors (Morin et al., 2018; Ali et al., 2019b). Previous studies have shown that climatic water availability and soil nutrients regulate forest productivity *via* shifting inter-intraspecific relationships, but their direction and intensity are closely related to species functional composition, succession stage, and stand age (Page-Dumroese et al., 2010; Coomes et al., 2014; Guo and Ren, 2014; Ouyang et al., 2019). For instance, it has been confirmed that the positive responses between productivity to mean annual precipitation and soil fertility are stronger in the mature forest than in young forest (Michaletz et al., 2014; Ouyang et al., 2019). More specifically, soil fertility decreased aboveground biomass in plantations indirectly *via* species diversity, large plants, and stand density, whereas the indirect effects were negligible in natural forests, indicating that the influences of abiotic factors are largely dependent on underlying biotic factors and thus context-dependent (Poorter et al., 2015; Ali et al., 2019b; Li et al., 2020). In plantations, abiotic factors regulated aboveground biomass *via* biotic factors. The strong indirect effect might be that the influence of initial anthropogenic inference on carbon sequestration decreases with stand age, and then the environmental resource availability intensifies the biotic process, which may decrease aboveground biomass (Subedi et al., 2012; Forrester, 2014; Wu et al., 2020). Previous studies have confirmed that with increasing stand age, there are stronger biotic interactions in plantation, such as self-thinning (Subedi et al., 2012; Guo and Ren, 2014). In an arid natural forest, the negligible indirect effect between soil fertility and aboveground biomass *via* biotic factors might be related to resource limitation (Guo et al., 2019; Li et al., 2020). Poor soil conditions limit plants’ growth process and hence weaken the indirect effect.

## Conclusions

A vast literature has explored the relationship between species diversity, stand structure, and forest function, but less so about the relationships among species diversity, large trees, and forest functions. Our study presents the first empirical results on the relationship among large plants, species diversity, stand density, and aboveground biomass in arid natural forests and plantations. This study highlights the importance of large plants to aboveground biomass in both natural forests and plantations along abiotic and biotic gradients, hence supporting the generality of the big-sized tree hypothesis and scaling theory. Although our results show that large plants, species diversity, and stand density increase aboveground biomass in both natural forests and plantations, the roles of climatic water availability and soil fertility seem to be differential in natural forests and plantations. We need to point out that the regulation of plant species diversity and large plants to aboveground biomass is dependent on spatial scale, indicating that numerous samplings across a large spatial scale for further exploration are necessary, particularly in sensitive and species-poor arid regions. Moreover, we argue that further work is still needed to explore the underlying role of plant species’ functional strategies, in terms of functional diversity and composition, in order to fully explore the underlying ecological mechanisms in natural forests and plantations.

## Data availability statement

The original contributions presented in the study are included in the article/**Supplementary Material**. Further inquiries can be directed to the corresponding authors.

## Author contributions

X-DY and AA developed the idea and designed the study. B-YY, YL, X-DY collected the data. B-YY analyzed the data and wrote the paper with full support from AA, YL, X-NZ, X-MH, and X-DY. All authors contributed to the article and approved the submitted version.

## Funding

This work was supported by the National Natural Science Foundation of China (Grant Nos. 41871031 and 31860111), Natural Science Foundation of Xinjiang (Grant No. 2017D01C080), and the Science and Technology Innovation 2025 Major Project of Ningbo City (Grant No. 20212ZDYF020049). Arshad Ali was supported by Hebei University (Grant No. 521100221033).

## Conflict of interest

The authors declare that the research was conducted in the absence of any commercial or financial relationships that could be construed as a potential conflict of interest.

## Publisher’s note

All claims expressed in this article are solely those of the authors and do not necessarily represent those of their affiliated organizations, or those of the publisher, the editors and the reviewers. Any product that may be evaluated in this article, or claim that may be made by its manufacturer, is not guaranteed or endorsed by the publisher.
