# Supplementary material for: Large plants enhance aboveground biomass in arid natural forest and plantation along differential abiotic and biotic conditions
Source: Front Plant Sci. 2022 Oct 13;13:999793. doi: 10.3389/fpls.2022.999793 (PMC9612956; doi:10.3389/fpls.2022.999793)
Supplement: Supplementary file 1 [file DataSheet_1.docx]

**Supporting Information**

**Large Plants Enhance Aboveground Biomass in Arid Natural Forest and Plantation Along Differential Abiotic and Biotic Conditions**

**Bai-Yu Yang^1,2^, Arshad Ali^3*^, Ming-Shan Xu^2^, Min-Sha Guan^1^, Yan Li^2^, Xue-Ni Zhang^2^, Xue-Min He^2^, Xiao-Dong Yang^1,2,4*^**

*^1^* *Department of Geography & Spatial Information Technology, Ningbo University, Ningbo 315211, China*

*^2^ Institute of Resources and Environment Science, Xinjiang University, Urumqi, China*

*^3^ Forest Ecology Research Group, College of Life Sciences, Hebei University, Baoding, 071002, Hebei, China*

*^4^ Institute of East China Sea, Ningbo University, Ningbo 315211, China*

***Corresponding author:** Arshad Ali arshadforester@gmail.com; and Xiao-Dong Yang xjyangxd@sina.com.

**Running title:** Drivers of aboveground biomass

**FIGURE S1** The distribution of climatic water availability (CAI) and sampling plot in the northwest arid region of China.


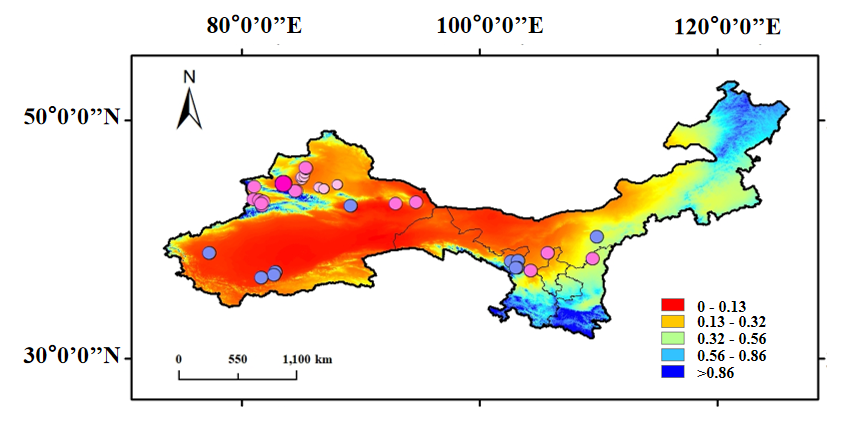


**FIGURE S2** The distribution of climatic water availability (CAI) and sampling plot in the northwest arid region of China.


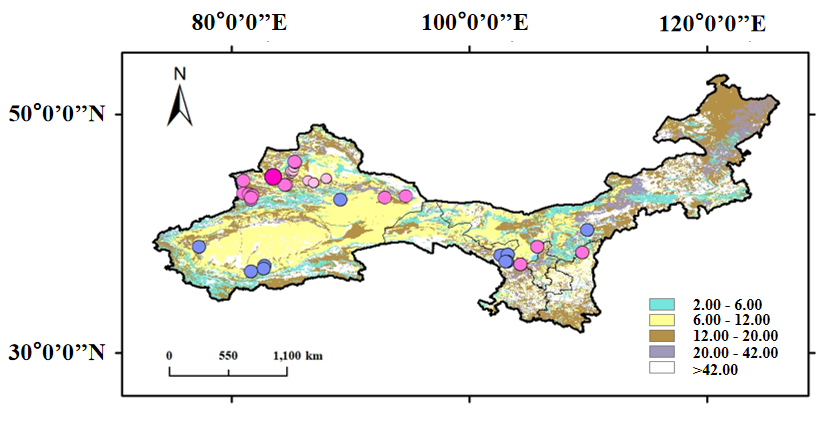


**TABLE S1** Allometric biomass equation of 25 species in arid forests

| **Number** | **Species name** | **Allometric growth equation** | **Reference** |
| --- | --- | --- | --- |
| 1 | *Picea schrenkiana* | AGB=0.047465712(DBH^2^×H)^0.88217^+0.00189147(DBH^2^×H)^1.03981^+0.01451443(DBH^2^×H)^0.78914^ | (Su, et al., 2007) |
| 2 | *Alhagi sparsifolia* | AGB=0.8645H+0.5262 | (Yang, 2019) |
| 3 | *Salsola arbuscula* | AGB=0.0011CA-0.3112 | (Buras, et al., 2012) |
| 4 | *Haloxylon ammodendron* | AGB=0.057754(DBH^2^×H)^0.849941^ | (Song and Hu 2011) |
| 5 | *Suaeda glauca* | AGB=0.8645H+0.5262 | (Yang, 2019) |
| 6 | *Calligonum mongolicum* | AGB=0.924(CA×H)^0.769^ | (Dang , et al., 2016) |
| 7 | *Nitraria tangutorum* | AGB=-0.278+0.32*D*_east-west_ -1.094*D*_north–south_ +8.951H | (Jia , et al., 2002) |
| 8 | *Tamarix chinensis* | AGB=e^6.385-90.007/lnCA^ | (Zhang and Wang 2019) |
| 9 | *Reaumuria soongonica* | AGB=21.4261lnCA-103.61 | (Zhao, et al., 2004) |
| 10 | *Nitraria sphaerocarpa* | AGB=0.8645H+0.5262 | (Zhao , et al., 2019) |
| 11 | *Populus euphratica* | AGB=128.09(DBH^2^×H)^0.6318^ | (Wang , et al., 2016) |
| 12 | *Kalidium foliatum* | AGB=0.8645H+0.5262 | (Yang, 2019) |
| 13 | *Halocnermum strobilaceum* | AGB=0.001(expCA)^0.013^ | (Zhang and Wang 2019) |
| 14 | *Lycium ruthenicum* | AGB=0.8645H+0.5262 | (Yang, 2019) |
| 15 | *Elaeagnus angustifolia* | AGB=0.01DBH^3^-0.219DBH^2^+6.314DBH+13.060 | (Ma , et al., 2016) |
| 16 | *Artemisia desertorum* | AGB=0.8645H+0.5262 | (Yang, 2019) |
| 17 | *Caragana korshinskii* | AGB=374.58+0.093(CA×H) | (Dang , et al., 2016) |
| 18 | *Ulmus glaucescens* | AGB=0.0146DBH^2.5837^+0.0303DBH^2.3445^+0.033DBH^1.7241^ | (Li 2006) |
| 19 | *Hedysarum scoparium* | AGB=694.43+0.047(CA×H) | (Dang , et al., 2016) |
| 20 | *Acanthophyllum pungens* | AGB=0.8645H+0.5262 | (Yang, 2019) |
| 21 | *Salix psammophila* | AGB=0.365(CA×H)^0.848^ | (Dang , et al., 2016) |
| 22 | *Populus tremula* | AGB=-0.004 DBH^2^+16.671DBH-104.12 | (Dong, et al., 2015) |
| 23 | *Caragana sinica* | AGB=0.8645H+0.5262 | (Yang, 2019) |
| 24 | *Halostachys caspica* | AGB=e^6.805-80.217/lnCA^ | (Zhang and Wang 2019) |
| 25 | *Salix matsudana* | AGB=41.147BD^2.3273^ | (Zhang and Wang 2019) |

**TABLE S2** The constituent species of large plants in natural forest and plantation. Frequency refers to the number of sampling plots in which large plants is formed from the related species.

| **Natural forest** | | | **Plantation** | | |
| --- | --- | --- | --- | --- | --- |
| Number | Latin name | Frequency | Number | Latin name | Frequency |
| 1 | *Haloxylon ammodendron* | 33 | 1 | *Haloxylon ammodendron* | 9 |
| 2 | *Populus euphratica* | 29 | 2 | *Elaeagnus angustifolia* | 7 |
| 3 | *Reaumuria soongonica* | 10 | 3 | *Tamarix chinensis* | 6 |
| 4 | *Picea schrenkiana* | 10 | 4 | *Hedysarum scoparium* | 3 |
| 5 | *Alhagi sparsifolia* | 7 | 5 | *Artemisia desertorum* | 3 |
| 6 | *Tamarix chinensis* | 6 | 6 | *Ulmus glaucescens* | 3 |
| 7 | *Hedysarum scoparium* | 6 | 7 | *Salix psammophila* | 2 |
| 8 | *Kalidium foliatum* | 6 | 8 | *Populus euphratica* | 1 |
| 9 | *Nitraria tangutorum* | 5 | 9 | *Caragana korshinskii* | 1 |
| 10 | *Nitraria sphaerocarpa* | 4 | 10 | *Calligonum mongolicum* | 2 |
| 11 | *Calligonum mongolicum* | 3 |  |  |  |
| 12 | *Artemisia desertorum* | 3 |  |  |  |
| 13 | *Salix psammophila* | 3 |  |  |  |
| 14 | *Lycium ruthenicum* | 2 |  |  |  |
| 15 | *Caragana sinica* | 1 |  |  |  |
| 16 | *Salsola arbuscula* | 1 |  |  |  |
| 17 | *Halostachys caspica* | 1 |  |  |  |

**TABLE S3** Direct, indirect and total standardized effects of predictors and mediators on aboveground biomass in natural forests, based on the structural equation model (see FIGURE3a). Significant effects (*P* < 0.05) are indicated in bold. Abbreviations for variables are explained in

| Response variable | Mediator variable 1 | Mediator variable 2 | Predictor variable | Effect | Path label | Beta | S.E. | *z*-value | *p*-values |
| --- | --- | --- | --- | --- | --- | --- | --- | --- | --- |
| Large plants | - | - | CAI | Direct | a | 0.25 | 0.23 | 1.84 | 0.07 |
| Species diversity | - | - | CAI | Direct | b | -0.70 | 0.05 | -8.58 | **0.00** |
| Stand density | - | - | CAI | Direct | c | -0.47 | 0.13 | -4.49 | **0.00** |
| Large plants | - | - | Soil fertility | Direct | d | -0.04 | 0.18 | -0.43 | 0.67 |
| Species diversity | - | - | Soil fertility | Direct | e | -0.10 | 0.05 | -1.27 | 0.21 |
| Stand density | - | - | Soil fertility | Direct | f | 0.11 | 0.14 | 1.03 | 0.30 |
| Large plants | - | - | Species diversity | Direct | g | -0.21 | 0.40 | -1.51 | 0.13 |
| Large plants | - | - | Stand density | Direct | h | -0.37 | 0.15 | -3.44 | **0.00** |
| AGB | - | - | Large plants | Direct | i | 1.10 | 0.25 | 10.35 | **0.00** |
| AGB | - | - | Species diversity | Direct | j | 0.19 | 0.62 | 2.15 | **0.03** |
| AGB | - | - | Stand density | Direct | k | 0.65 | 0.26 | 8.53 | **0.00** |
| AGB | - | - | CAI | Direct | l | 0.09 | 0.36 | 1.04 | 0.30 |
| AGB | - | - | Soil fertility | Direct | m | -0.33 | 0.27 | -5.53 | **0.00** |
| AGB | Large plants | - | CAI | Indirect | ai | 0.27 | 0.62 | 1.81 | 0.07 |
| AGB | Species diversity | - | CAI | Indirect | bj | -0.13 | 0.26 | -2.09 | **0.04** |
| AGB | Stand density | - | CAI | Indirect | ck | -0.30 | 0.32 | -3.97 | **0.00** |
| AGB | Large plants | - | Soil fertility | Indirect | di | -0.05 | 0.48 | -0.43 | 0.67 |
| AGB | Species diversity | - | Soil fertility | Indirect | ej | -0.02 | 0.08 | -1.09 | 0.28 |
| AGB | Stand density | - | Soil fertility | Indirect | fk | 0.07 | 0.32 | 1.02 | 0.31 |
| AGB | Large plants | - | Species diversity | Indirect | gi | -0.23 | 1.07 | -1.50 | 0.13 |
| AGB | Large plants | - | Stand density | Indirect | hi | -0.40 | 0.41 | -3.28 | **0.00** |
| AGB | Species diversity | Large plants | CAI | Indirect | bgi | 0.16 | 0.44 | 1.48 | 0.14 |
| AGB | Stand density | Large plants | CAI | Indirect | chi | 0.19 | 0.29 | 2.65 | **0.01** |
| AGB | Species diversity | Large plants | Soil fertility | Indirect | egi | 0.02 | 0.11 | 0.97 | 0.33 |
| AGB | Stand density | Large plants | Soil fertility | Indirect | fhi | -0.04 | 0.20 | -0.98 | 0.33 |
| AGB | Direct + Indirect | Direct + Indirect | Species diversity | Total | j | 0.19 | 0.62 | 2.15 | **0.03** |
| AGB | Direct + Indirect | Direct + Indirect | Stand density | Total | k+hi | 0.25 | 0.40 | 2.05 | **0.04** |
| AGB | Direct + Indirect | Direct + Indirect | CAI | Total | bj+ck+ chi | -0.25 | 0.36 | -2.85 | **0.00** |
| AGB | Direct + Indirect | Direct + Indirect | Soil fertility | Total | m | -0.33 | 0.27 | -5.53 | **0.00** |

Note*: non-significant effects of top 20% large plants, Hs, SD, CAI on AGB were excluded when we calculated the total effects.

**TABLE S4** Direct, indirect and total standardized effects of predictors and mediators on aboveground biomass in plantations, based on the structural equation model (see **Figure 3b**). Significant effects (*P* < 0.05) are indicated in bold. Abbreviations for variables are explained in **Table S3**.

| Response variable | Mediator variable 1 | Mediator variable 2 | Predictor variable | Effect | Path label | Beta | S.E. | *z*-value | *p*-values |
| --- | --- | --- | --- | --- | --- | --- | --- | --- | --- |
| Large plants | - | - | CAI | Direct | a | -0.63 | 0.05 | -6.15 | **0.00** |
| Species diversity | - | - | CAI | Direct | b | 0.14 | 0.04 | 0.83 | 0.41 |
| Stand density | - | - | CAI | Direct | c | 0.03 | 0.08 | 0.17 | 0.86 |
| Large plants | - | - | Soil fertility | Direct | d | -0.28 | 0.14 | -2.37 | **0.02** |
| Species diversity | - | - | Soil fertility | Direct | e | -0.40 | 0.09 | -2.43 | **0.02** |
| Stand density | - | - | Soil fertility | Direct | f | 0.22 | 0.19 | 1.22 | 0.22 |
| Large plants | - | - | Species diversity | Direct | g | 0.45 | 0.26 | 3.66 | **0.00** |
| Large plants | - | - | Stand density | Direct | h | -0.40 | 0.13 | -3.53 | **0.00** |
| AGB | - | - | Large plants | Direct | i | 0.83 | 0.66 | 4.82 | **0.00** |
| AGB | - | - | Species diversity | Direct | j | 0.55 | 0.89 | 5.04 | **0.00** |
| AGB | - | - | Stand density | Direct | k | 0.24 | 0.42 | 2.40 | **0.02** |
| AGB | - | - | CAI | Direct | l | 0.13 | 0.25 | 1.04 | 0.30 |
| AGB | - | - | Soil fertility | Direct | m | 0.52 | 0.41 | 5.73 | **0.00** |
| AGB | Large plants | - | CAI | Indirect | ai | -0.53 | 0.27 | -3.81 | **0.00** |
| AGB | Species diversity | - | CAI | Indirect | bj | 0.08 | 0.18 | 0.82 | 0.41 |
| AGB | Stand density | - | CAI | Indirect | ck | 0.01 | 0.08 | 0.17 | 0.86 |
| AGB | Large plants | - | Soil fertility | Indirect | di | -0.24 | 0.51 | -2.13 | **0.03** |
| AGB | Species diversity | - | Soil fertility | Indirect | ej | -0.22 | 0.46 | -2.19 | **0.03** |
| AGB | Stand density | - | Soil fertility | Indirect | fk | 0.05 | 0.22 | 1.09 | 0.28 |
| AGB | Large plants | - | Species diversity | Indirect | gi | 0.37 | 1.04 | 2.93 | **0.00** |
| AGB | Large plants | - | Stand density | Indirect | hi | -0.33 | 0.49 | -2.86 | **0.00** |
| AGB | Species diversity | Large plants | CAI | Indirect | bgi | 0.05 | 0.13 | 0.80 | 0.42 |
| AGB | Stand density | Large plants | CAI | Indirect | chi | -0.01 | 0.12 | -0.17 | 0.86 |
| AGB | Species diversity | Large plants | Soil fertility | Indirect | egi | -0.15 | 0.37 | -1.87 | 0.06 |
| AGB | Stand density | Large plants | Soil fertility | Indirect | fhi | -0.07 | 0.30 | -1.12 | 0.26 |
| AGB | Direct + Indirect | Direct + Indirect | Species diversity | Total | j+gi | 0.92 | 0.98 | 7.62 | **0.00** |
| AGB | Direct + Indirect | Direct + Indirect | Stand density | Total | k+hi | -0.09 | 0.47 | -0.84 | 0.40 |
| AGB | Direct + Indirect | Direct + Indirect | CAI | Total | ai | -0.53 | 0.27 | -3.81 | **0.00** |
| AGB | Direct + Indirect | Direct + Indirect | Soil fertility | Total | m+ej+egi+di | -0.09 | 0.84 | -0.50 | 0.62 |

**FIGURE S3** The bivariate relationships between exogenous and endogenous variables used in structural equation model of natural forest. Solid lines represent significant paths (*P* < 0.05). Abbreviations: CAI, climatic aridity index as a representative of climatic water availability; SD, stand density; Hs, species diversity; Tall-H, tall-stature plants (top 20%); big-C, big-crown plants (top 20%); AGB, aboveground biomass; std, standardized; ln, natural-log.

**
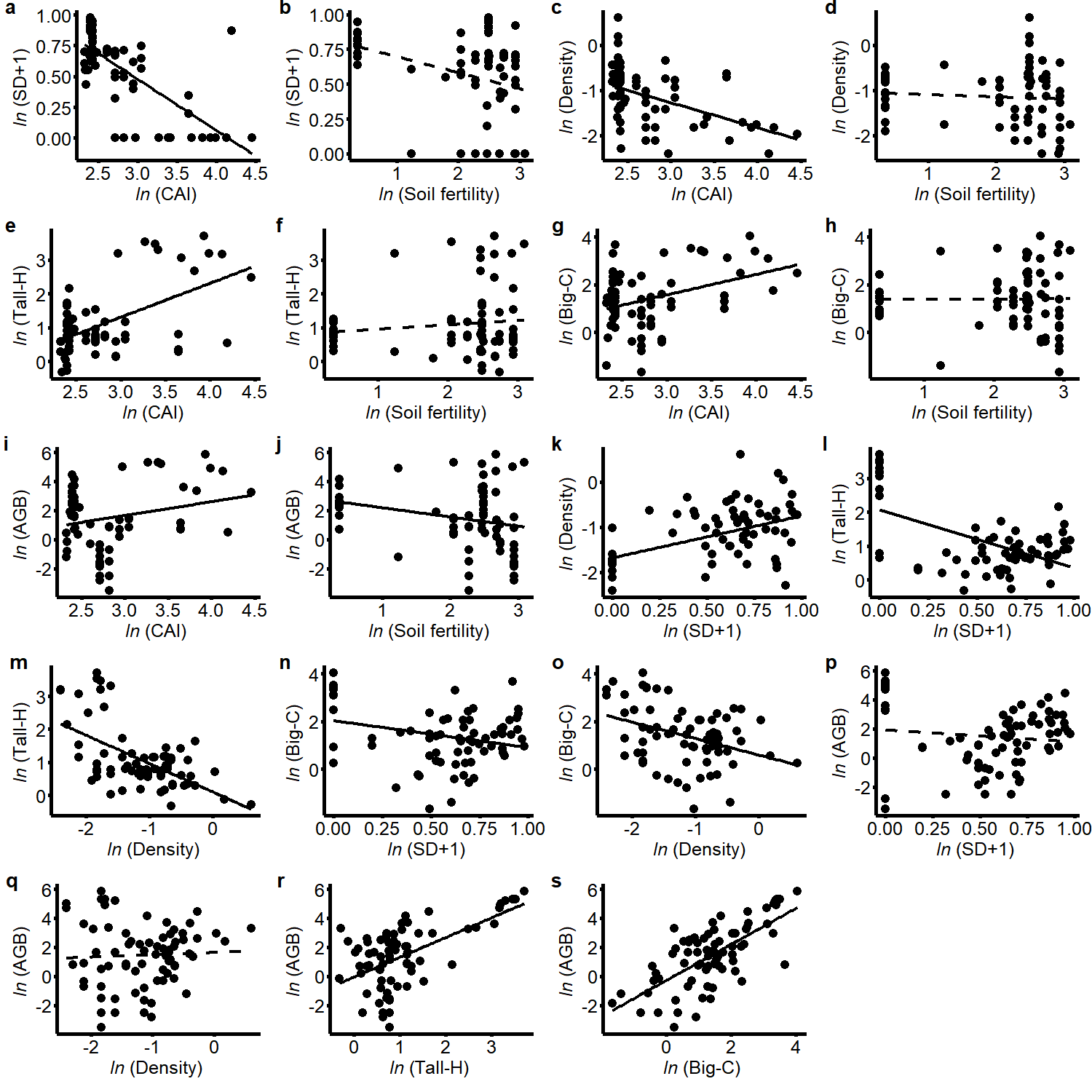
**

**FIGURE S4** The bivariate relationships between exogenous and endogenous variables used in structural equation model of plantation. Solid lines represent significant paths (*P* < 0.05). Abbreviations are explained in **Figure S3**.

**
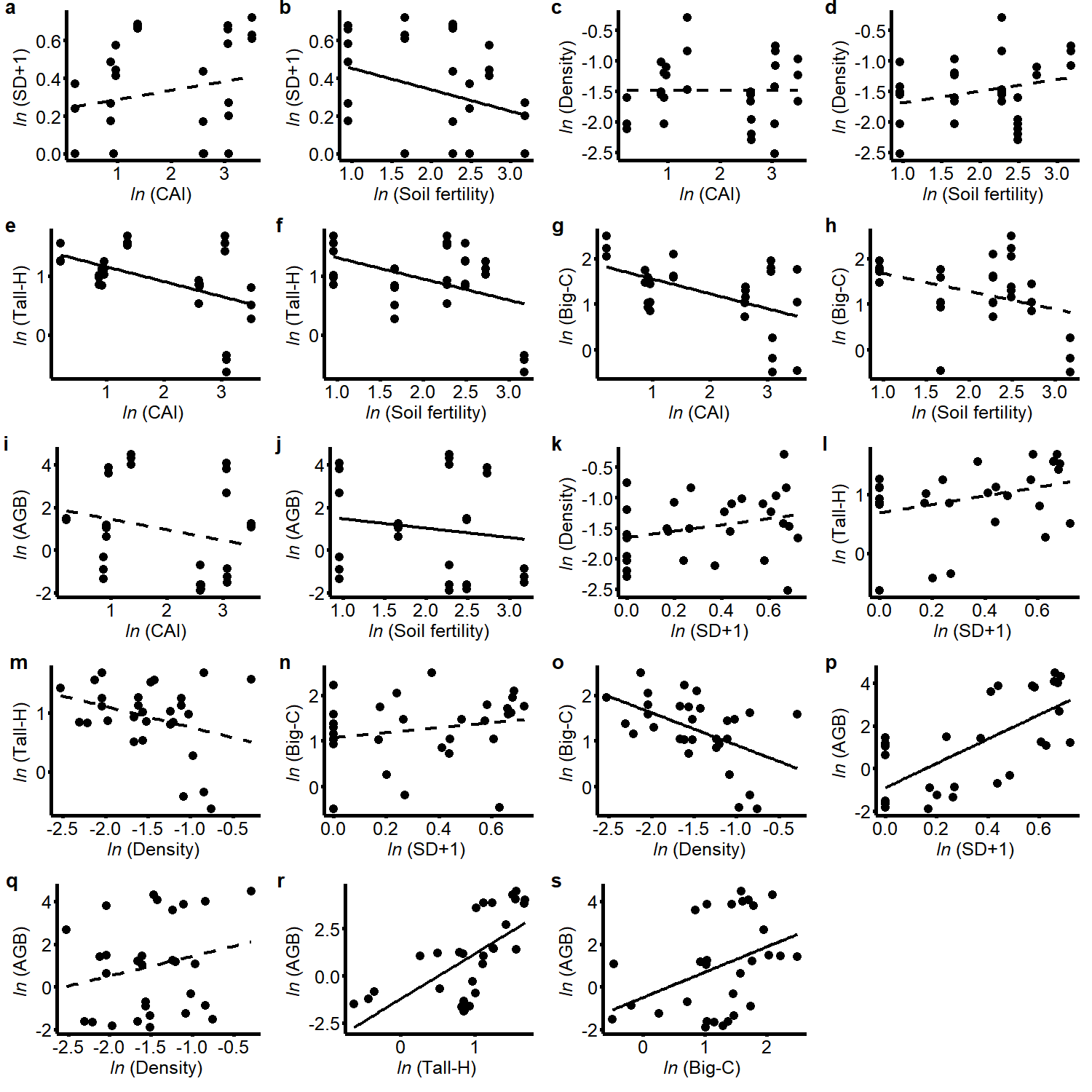
**

**References**

Buras, A., Wucherer, W., Zerbe, S., Noviskiy, Z., Muchitdinov, N., Shimshikov, B., et al. (2012). Allometric variability of Haloxylon species in Central Asia. *For. Ecol. Manag.* 274**,** 0-9. doi: 10.1016/j.foreco.2012.02.023.

Dang , X. H., Gao , Y., Yu , Y., Meng , Z. J. and Liu , Y. (2016). The biomass estimation models for eight desert shrub species in northern edge of the Hobq Desert. *J Arid Land Resour. Environ.* 030(005)**,** 168-174. doi: 10.13448/j.cnki.jalre.2016.165.

Dong, Y. F., Kuang , X. X., Qin , N. H., Wang , W. D., Qin , G. H. and Jiang , Y. Z. (2015). Effects of Initial Planting Spacing on Growth and Above-ground Biomass of *Populous* Plantation. *J Northeast For Univ*. 43.09, 30-33. doi:10.13759/j.cnki.dlxb.20150721.005.

Jia , B. Q., Cai , T. J., Gao , Z. H. and Ding, F. (2002). Biomass Forcast Models of Nitraria Tangutorum Shrub in Sand Dune. *J Arid Land Resour. Environ.* 016(1)**,** 96-99.

Li , G. (2006). Biomass, Net Primary Productivity,and Ecological Restoration Space Forest Grassland Ecosystem Hunshandake Sandy Land.

Ma , L. Q., Yang , F. Q., Feng , D. L. and Xu , L. (2016). Gansu Minqin Desert Elaeagnus angustifolia Biomass Density Study. *J Gansu For.Sci Technol.* (4). doi: 10.3969/j.issn. 1006-0960.2016.04.002.

Song , Y. Y. and Hu , J. X. (2011). Estimation Models of the A boveg round Biomass of Haloxy lon ammodendron in the Different Ecotype of Gurbantunggut Desert. *J Northwest For Univ*. 26(2),31-37.

Su, H., Sang, W., Wang, Y. and Ma, K. (2007). Simulating Picea schrenkiana forest productivity under climatic changes and atmospheric CO2 increase in Tianshan Mountains, Xinjiang Autonomous Region, China. *For. Ecol. Manag.* 246(2-3)**,** 0-284. doi: 10.1016/j.foreco.2007.04.010.

Wang , X. Y., Xu , H. L., Pan, C. D., Ling , H. B. and Fan , K. Y. (2016). Spatial distribution of *populus euphratica* biomass in the lower reaches of Tarim river. *Acta Botanica Boreali-Occidentalia Sinica*. (11)**,** 2314-2321. doi: 10.7606/j.issn.1000-4025.2016.11.2314.

Yang, S. (2019). Estimate of Aboveground Biomass of Different Types of Grassland.

Zhang , D. D. and Wang , X. M. (2019). Estimating aboveground biomass of typical plant species in the oasis-desert transition zone of northern Tarim Basin. *Chinese J Ecol.* 38(10)**,** 3211-3220. doi: 10.13292 /j.1000－4890.201910.024.

Zhao, C. Y., Song, Y. D., Wang, Y. C. and Jiang, P. A. (2004). Estimation of aboveground biomass of desert plants. *Chinese J Appl Ecol* . 15(1)**,** 49-52. doi: 10.13287/j.1001-9332.2004.0011.

Zhao , M. Y., Sun , W., Luo , Y. K., Liang , C. Z. and Li , Z. Y. (2019). Models for Estimating the Biomass of 26 Temperate Shrub Species in Inner Mongolia，China. *Arid Zone Res.* (5). doi: 10.13866/j.azr.2019.05.20.
