# Supplementary material for: Large plants enhance aboveground biomass in arid natural forest and plantation along differential abiotic and biotic conditions
Source: Front Plant Sci. 2022 Oct 13;13:999793. doi: 10.3389/fpls.2022.999793 (PMC9612956; doi:10.3389/fpls.2022.999793)
Supplement: Supplementary file 3 [file DataSheet_3.docx]

Code in R

data_a<-read.csv(file.choose(),header=T) # natural forest

data_b<-read.csv(file.choose(),header=T) # plantation

# AGB = Aboveground biomass

# LP = Large plants

# TH = Tall Height

# BC = Big Crown

# Hs = species diversity

# SD = stand density

# CAI = Climatic aridity index

# SOIL = Soil fertility

# all data has Natural-logarithm transformed and standardized

library(lavaan)

#Natural forest

modela1<-'

LP = ~ TH + BC

Hs ~ CAI + SOIL

SD ~ CAI + SOIL

LP ~ Hs + SD + CAI + SOIL

AGB ~ LP + Hs + SD + CAI + SOIL

SD~~Hs

'

fita1 <- sem(modela1,data=data_a)

summary(fita1, standardized=TRUE, rsq=TRUE)

fitMeasures(fita1,c("cfi","gfi","srmr"))

modelaa1<-'

SD~~Hs

LP = ~TH + BC

LP ~ a*CAI

Hs ~ b*CAI

SD ~ c*CAI

LP ~ d*SOIL

Hs ~ e*SOIL

SD ~ f*SOIL

LP ~ g*Hs

LP ~ h*SD

AGB ~ i*LP

AGB ~ j*Hs

AGB ~ k*SD

AGB ~ l*CAI

AGB ~ m*SOIL

ai := a*i

bj := b*j

ck := c*k

di := d*i

ej := e*j

fk := f*k

gi := g*i

hi := h*i

bg := b*g

ch := c*h

eg := e*g

fh := f*h

bgi := bg*i

chi := ch*i

egi := eg*i

fhi := fh*i

indirectSD := hi

indirectCAI := bj + ck +chi

totalLP := i

totalHs := j

totalSD := k + hi

totalCAI := bj + ck +chi

totalSOIL := m

'

fitaa1 <- sem(modelaa1, data =data_a)

summary(fitaa1, standardized=TRUE, rsq=TRUE)

fitMeasures(fitaa1,c("cfi","gfi","srmr"))

#Plantation

modelb1<-'

LP = ~ TH + BC

Hs ~ CAI + SOIL

SD ~ CAI + SOIL

LP ~ Hs + SD + CAI + SOIL

AGB ~ LP + Hs + SD + CAI + SOIL

SD~~Hs

'

fitb1 <- sem(modelb1,data=data_b)

summary(fitb1, standardized=TRUE, rsq=TRUE)

fitMeasures(fitb1,c("cfi","gfi","srmr"))

modelbb1<-'

SD~~Hs

LP = ~TH + BC

LP ~ a*CAI

Hs ~ b*CAI

SD ~ c*CAI

LP ~ d*SOIL

Hs ~ e*SOIL

SD ~ f*SOIL

LP ~ g*Hs

LP ~ h*SD

AGB ~ i*LP

AGB ~ j*Hs

AGB ~ k*SD

AGB ~ l*CAI

AGB ~ m*SOIL

ai := a*i

bj := b*j

ck := c*k

di := d*i

ej := e*j

fk := f*k

gi := g*i

hi := h*i

bg := b*g

ch := c*h

eg := e*g

fh := f*h

bgi := bg*i

chi := ch*i

egi := eg*i

fhi := fh*i

indirectHs := gi

indirectSD := hi

indirectCAI := ai

indirectSOIL := ej + egi + di

totalLP := i

totalHs := j + gi

totalSD := k + hi

totalCAI := ai

totalSOIL := m + ej + egi + di

'

fitbb1 <- sem(modelbb1, data =data_b)

summary(fitbb1, standardized=TRUE, rsq=TRUE)

fitMeasures(fitbb1,c("cfi","gfi","srmr"))
